# Maternal Hypertension, Advanced Doppler Haemodynamics and Therapeutic Precision: Principles and Illustrative Cases

**DOI:** 10.1007/s11906-020-01060-2

**Published:** 2020-07-13

**Authors:** Rob A. Phillips, Z. Ma, B. Kong, L. Gao

**Affiliations:** 1 The Critical Care Research Group, Department of Medicine, The University of Queensland, Brisbane, Australia; 2 Department of Maternal Intensive Care Medicine Unit, Shandong Maternal and Child Health Hospital, Jinan, Shandong China

**Keywords:** Maternal hypertension, Advanced haemodynamics, Precise management, Pre-eclampsia

## Abstract

**Purpose of Review:**

Maternal hypertension is a common and serious condition associated with increased maternal and foetal morbidity and mortality, with early detection and management improving outcomes.

**Recent Findings:**

Blood pressure (BP) changes of pre-eclampsia are defined after 20 gestational weeks, while haemodynamic changes can be detected at 5–11 weeks using a specialised non-invasive Doppler stroke volume (SV) monitor. Thus, advanced haemodynamic monitoring allows for physiologically precise identification of circulatory abnormalities, and implementation of appropriate therapy within the first trimester. We measured the oscillometric BP and advanced haemodynamics (USCOM 1A) of 3 unselected women with singleton pregnancies, consecutively listed for therapeutic induction for maternal hypertension at 32–41 weeks gestational age. While the BP’s of the patients varied, it was the haemodynamics, particularly SV, cardiac output, systemic vascular resistance, Smith Madigan Inotropy Index, and oxygen deliver, that identified differing patterns of circulatory dysfunction, therapeutic objectives, and predicted post-partum complications of the mother and child.

**Summary:**

First trimester screening of maternal haemodynamics may allow for earlier detection of circulatory derangements, selection of patient precise interventions, and improved maternal-foetal outcomes.

## Introduction

Hypertension in pregnancy is a common and serious condition associated with significantly increased maternal and foetal morbidity and mortality, as well as increased cost of care [1–5]. While there has been an increasing awareness and focus on maternal hypertension, morbidity and mortality outcomes remain persistently poor despite promotion of widespread blood pressure (BP)-guided management protocols [6•, 7•, 8].

The heart and vessels function to deliver oxygen and substrates to the cells and are regulated by the autonomic nervous system (ANS) and its sensors, predominantly baroreceptors, which act to preserve BP. As Guyton demonstrated, BP is the product of cardiac and vascular function with its interdependence described by the equation [9].

$$ {\displaystyle \begin{array}{c}\mathrm{MAP}=\mathrm{CO}\times \mathrm{SVR}\\ {}\mathrm{or}\\ {}\mathrm{MAP}=\left(\mathrm{SV}\times \mathrm{HR}\right)\times \mathrm{SVR}\end{array}} $$

where MAP = mean arterial pressure (mmHg), SVR = systemic vascular resistance (dynes.s.cm-5) SV = stroke volume (mls), HR = heart rate (bpm) and CO = cardiac output (l/min). This distinction of pressure and flow remains incompletely appreciated in the clinical assessment of the circulation and its management, particularly in hypertension. Therefore, in the compensatory or pre-hypertensive phase of circulatory dysfunction, small changes in SV, CO, and SVR can be masked by ANS modulated compensation to baroreceptor determined set points (Table 1). This mathematical coupling ensures that normal BP may mask underlying circulatory dysfunction in the compensated phase. With increasing dysfunction, the compensatory mechanism is exceeded and the BP rises in hypertension and decreases in hypotension (Table 2). However, even in the decompensated or expressed phase, with an elevated BP, the distinction between SV and or SVR dysfunction cannot be resolved and appropriate physiologic targeting of therapies is not possible.

**Table 1:**
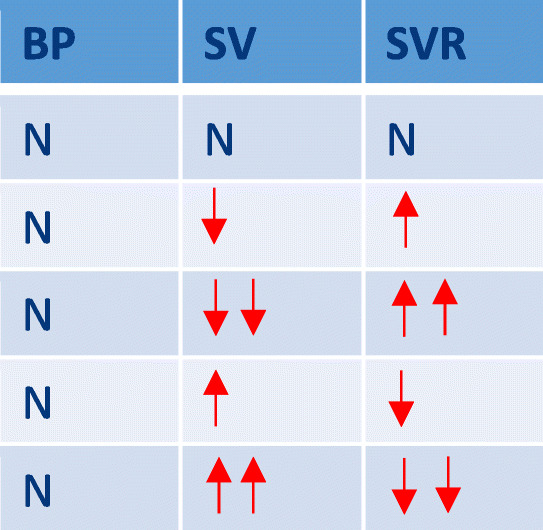
Simplified relationship of SV and SVR in normotensive subjects demonstrating the potential for masking of circulatory dysregulation in the compensatory or “pre-hypertensive” phase of hypertension (N=normal, =increased, =greatly increased, =decreased, =greatly decreased)

**Table 2:**
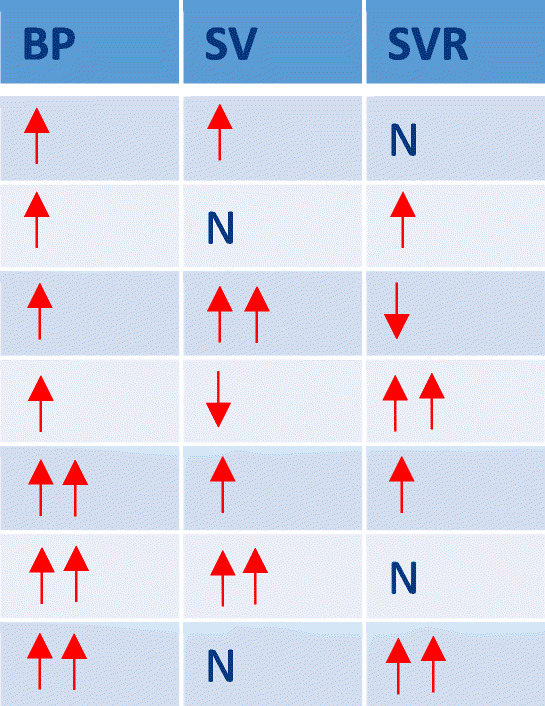
Simplified relationship of SV and SVR in hypertensive subjects demonstrating circulatory dysregulation in the decompensatory or expressed phase of hypertension (N=normal, =increased, =greatly increased, =decreased, =greatly decreased)

While BP is both a poor surrogate of cardiovascular function and DO_2_ [10], and a poor guide for therapy, direct measurement of SV and SVR provides objective measures of circulatory dysfunction and identifies physiologic targets for therapy in normotensive and hypertensive subjects.

In normal pregnancy oxygen consumption, VO2 in ml/min is increased, and oxygen delivery, DO_2_ in ml/min, is upregulated to maintain adequate utero-placental perfusion to support the growing foetus. This upregulation involves general vasodilation, predominantly in the 5th to 16th weeks [11], resulting in a reduced SVR [12], and an increased SV, CO, SMII and DO_2_. Fluid retention also increases the total blood volume, and the preload and consequently the SV, CO, SMII and DO_2_. This paradoxic decrease in SVR and increase in SV generally preserves the BP throughout a normal pregnancy, even as DO_2_ is upregulated [13].

Maternal hypertension is thought to have its pathogenesis in the maladaptation of the spiral arteries in the uterus and placenta leading to failure of normal adaptive vasodilation [12]. This maladaptation results in a relatively increased maternal SVR and a compensatory decrease in SV, CO, DO_2_ and SMII, resulting in relative placental-foetal hypoxia. Tiralongo observed that “As the normal maternal hemodynamic adaptation to pregnancy is an increase in SV, CO and SMII, accompanied by a reduced SVR, it is unsurprising that a decreased SV, CO and SMII and an accompanying increase in SVR results in poor maternal-fetal outcomes” [14••].

The range of normal maternal haemodynamics varies considerably throughout pregnancy [15, 16], with obesity [17, 18], and with the degree and origin of any dysfunction, and in response to ANS compensation [15, 19••, 20, 21]. This creates a moving target for therapy and limits the potential for simple protocolised interventions, an observation supported by the relative ineffectiveness of current BP-guided therapeutic guidelines [6•, 7•, 8]. A shift to physiologic, patient precise therapy is necessary to improve the effectiveness of maternal hypertension management.

**Fig. 1:**
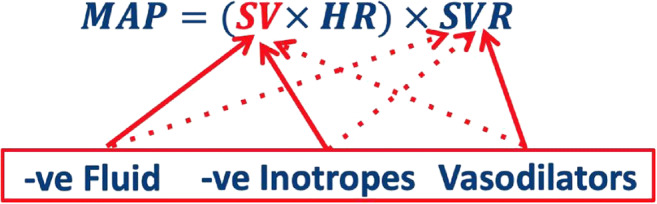
Relationship of BP, and the haemodynamic variables of SV and SVR [9], demonstrating the direct (continuous lines) and ANS-regulated (dashed lines) action of antihypertensive therapies. Hypertensive therapies do not act directly on BP

Hypertension is treated by reducing CO, SVR or both. CO is reduced by reducing preload, and thus SV, with a diuretic, and/or, by reducing HR and myocyte contractility, or by decreasing SVR using a vasodilator (Fig. 1) [6•]. Therefore, direct measurement of the SV and SVR is essential for early detection of circulatory dysregulation and appropriate choice of, and monitoring of, therapeutic interventions, particularly in the early latent or compensated phase of hypertension.

The non-invasive USCOM 1A Doppler monitor (Uscom Limited, Sydney Australia) has recently been validated for advanced point of care monitoring of haemodynamics in pregnancy [22–24]. The device directly measures transvalvular ventriculoarterial blood flow and generates beat to beat measures of SV, CO, SVR, SMII and DO_2_, plus 20 additional advanced haemodynamic parameters [25]. SMII is a novel measure of ventricular inotropy based on quantitation of the potential and kinetic energy of the circulation indexed to the time of ventricular ejection [26] and is an improved index of total cardiovascular load in W/m^2^. The USCOM 1A has been proven to be reliable, reproducible and more sensitive than bio-impedance [18, 27••, 28•, 29], and more feasible to use than echocardiography [27••], with measurements acquired in a few minutes [14••]. Established normal haemodynamic reference values across pregnancy [14••, 19••] and a high sensitivity to SV change ensure early detection of circulatory dysregulation and accurate identification of appropriate therapy and monitoring of therapeutic response. Using this method, Tiralonga et al. identified the haemodynamic changes of pre-eclampsia at 5 to 11 weeks [14••], while Valensise et al. demonstrated that a low CO and high SVR, > 1069 dynes s cm^−5^, at the end of pregnancy are associated with higher risks of foetal distress and maternal complications [30] and preterm delivery [21]. Tiralonga also demonstrated that 2 weeks of treatment of women with maternal hypertension and foetal growth restriction using NO donors and volume expansion at 30 weeks gestational age significantly changed SV, CO and SVR and improved maternal and foetal outcomes [31••]. While the diagnostic criteria for pre-eclampsia is a BP > 140/90 after twenty gestational weeks [6•, 7•], the adoption of normal haemodynamic reference values is more intuitive and may provide diagnostic and therapeutic guidance at 5–11 weeks, and therefore improve outcomes [20, 31•, 32, 33].

Maternal hypertension is often treated with induction and Caesarean section [6•], which may increase risks in consecutive pregnancies [34]. Early detection of maternal hypertension and precision management may facilitate earlier and improved management and reduce the incidence of these interventions, reducing risks in first and successive pregnancies.

The monitoring of maternal haemodynamics has demonstrated promising results for the early identification and patient precise targeting of treatment of maternal hypertension [31•, 35, 36, 37•]. We present a series of three consecutive case studies to demonstrate the principles supporting this promising and rapidly evolving clinical approach to maternal hypertension.

## Method

Contemporaneously measured BP and Doppler SV haemodynamic examinations were performed on three randomly selected women with singleton pregnancies at 32 to 41 weeks gestational age following informed consent. Participants were consecutively listed for therapeutic induction for maternal hypertension in the 24 h following testing at a maternal tertiary referral centre in a Chinese provincial hospital. Maternal haemodynamics were measured using the USCOM 1A MATERNAL haemodynamic monitor (Uscom Limited, Sydney, Australia) and BP measures acquired using routine brachial oscillometry (Omron, Japan), with all measures acquired 5 min following the subjects placed in the supine resting position. The BP and USCOM haemodynamic measures were collated and compared. Normal values for pregnant haemodynamics were referenced from Vinayagam et al. [19••]. Grading of maternal hypertension was according to the NICE guidelines (mild hypertension 140–149/90–99 mmHg, moderate hypertension 150–159/100–109 mmHg, and severe hypertension > 160/110 mmHg [6•]). Clinical observations and maternal and foetal outcomes post partum were assessed.

## Findings

### Case 1

Normal adaptive circulation

Thirty-two-year-old (160 cm, 75Kg, BSA 1.87 m^2^) with a normal BP of 119/65, MAP 83, and one prior normal pregnancy with mild late term hypertension, for induction under anaesthesia at 41 weeks

Normal vaginal delivery of a 4130-g baby at 41 weeks gestational age with an Apgar of 10. Returned from the ICU 1 day post parturition

**Fig. 2:**
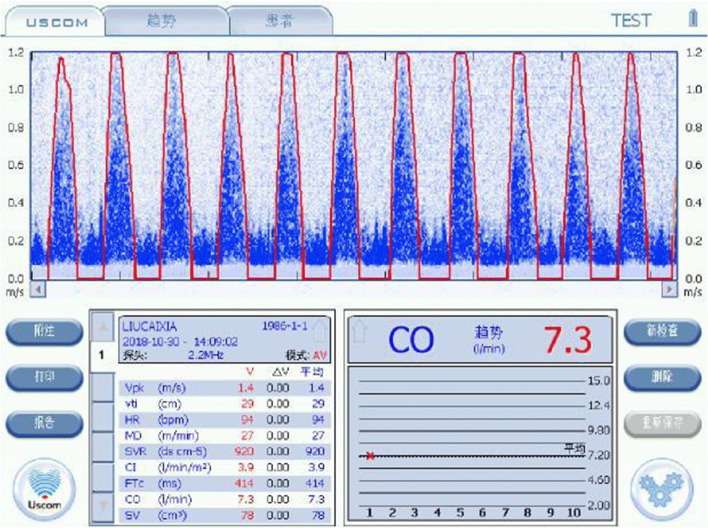
Normal high output adaptive circulation with increased SV, CO and DO_2_, with a normal SMII and decreased SVR

Summary—Normal adaptive haemodynamics with increased SV, CO, DO_2_ and SMII, with decreased SVR, and upregulated DO_2_ to meet increased VO2 of pregnancy (Fig. 2). Physiologically guided therapy—non-intervention with normal serial haemodynamic monitoring.

Outcome: uneventful birth and outcome.

### Case 2

Mild cardiogenic hypertension

Thirty-three-year-old (155 cm, 60.5Kg, BSA 1.65 m2, AV OTD 1.80) presenting with mild hypertension, 137/92 and MAP 107 mmHg and a history of one prior pregnancy complicated by mild hypertension. For planned induction at 36 weeks.

Normal vaginal delivery of a 2780-g baby at 32 gestational weeks with an Apgar score of 8. Mother and baby recovered quickly with an overnight observational ICU stay.

**Fig. 3:**
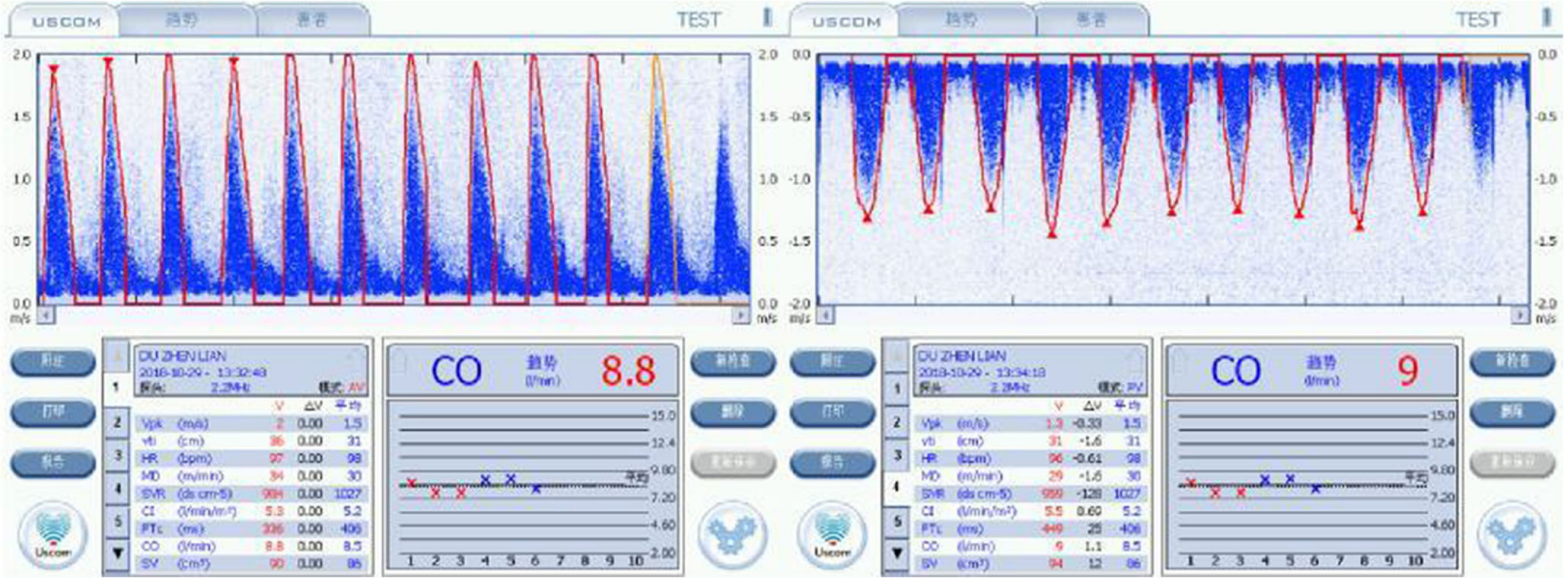
Aortic and pulmonary USCOM 1A output traces demonstrating moderately increased SV and CO across repeated measures, decreased SVR and moderately increased DO_2_

Summary—Mild cardiogenic hypertension (137/92 and MAP 107 mmHg) with moderately elevated SV, CO, HR, SMII and DO_2_, with a mildly decreased SVR (Fig. 3). To maintain the increased output, the cardiovascular system is overloaded despite a low normal SVR. Physiologically guided therapy would have targeted at reducing the SV using a diuretic. Outcome—At birth, the baby had a slightly decreased APGAR which quickly resolved, and both mother and child recovered after 24 h ICU observation.

### Case 3

Severe vasogenic hypertension with severely impaired cardiac function.

A 33-year-old obese mother (162 cm, 95.5 kg, BSA 2.14 m^2^) with 3 prior pregnancy’s complicated by moderate hypertension, presented at 32 weeks gestational age with severe hypertension, BP 165/95 (MAP = 118) and increasing oedema and leg pitting. The patient was scheduled for a therapeutic induction at 32 weeks gestational age.

Caesarean section delivered small for dates baby, 1550 g at 32 weeks gestational age with an Apgar score at 1 min of 9, at 5 min 7, and at 10 min 9, followed by a 3-day ICU stay.

**Fig. 4:**
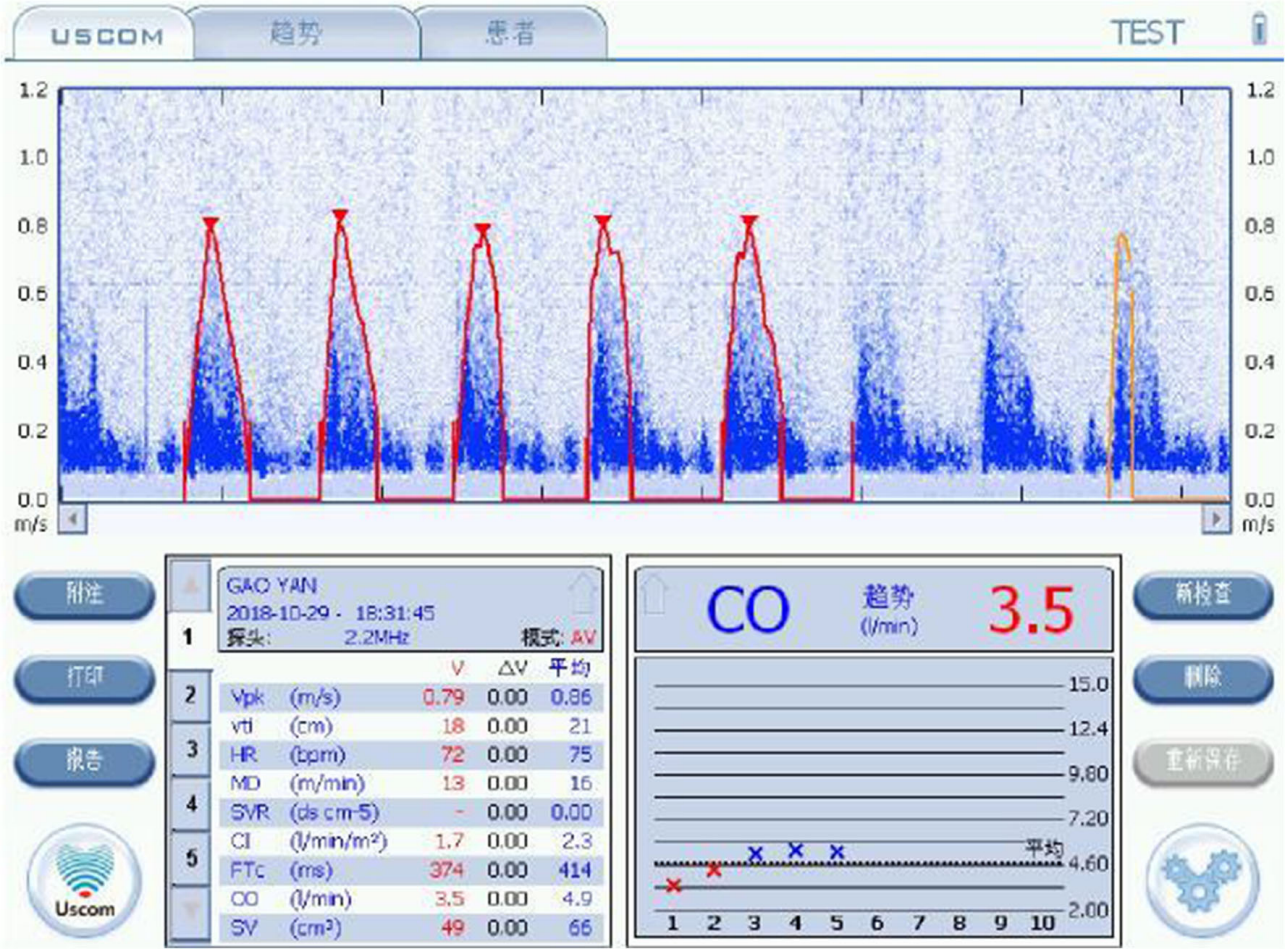
Case 3 aortic CO profile demonstrating severe vasogenic hypertension, with a severely elevated SVR, 2332 dyne s cm^−5^ (Normal 800–1000 dyne s cm^−5^), and severe impairment of SV of 46 ml (> 79 ml normal), CO of 3.5 L/min (normal 7–8 L/min) and SMII of 1.1 W/m^2^ (normal 1.1–2 W/m^2^), while DO_2_ was severely impaired, delivering approximately 60% of normal (594 v 900 ml/m^2^)

Summary—Severe vasogenic hypertension, with a severely elevated SVR (2332 v normal 1100 dyne s cm^−5^). Additionally, there was severe impairment of the SV (46 v normal 79 ml), CO (4 v 6.9 L/min), SMII (1.1 v 2.0 W/m^2^) and, importantly, DO_2_ was approximately 60% of normal (594 v 900 ml/m^2^) (Fig. 4).

Physiologically guided therapy would have targeted reducing the SVR with a vasodilator, and an inotrope to stimulate the LV function.

Outcome—The baby, after Caesarean birth, had a depressed 5 point APGAR score, which recovered over the first hour, while the mother required 3 days of ICU care. It is probable that this significant haemodynamic dysfunction would have been expressed in the first trimester and would have been detected by haemodynamic monitoring. This may have led to improved physiologically targeted management and a more stable haemodynamic course through pregnancy and improved outcome.

### Advanced Haemodynamics

**Fig. 5:**
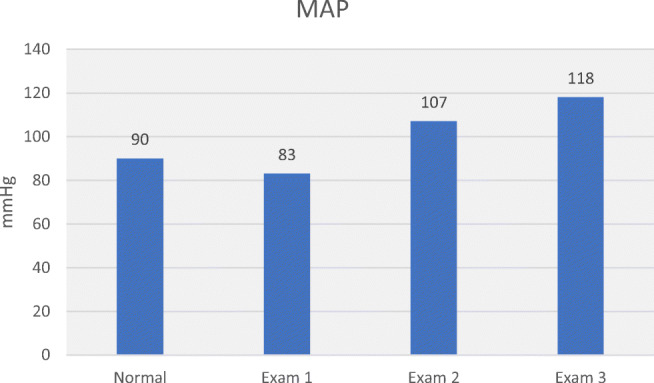
Normal reference MAP, normotensive pregnancy (Exam 1), mild hypertension (Exam 2) and severe hypertensive (Exam 3) [19••]

**Fig. 6:**
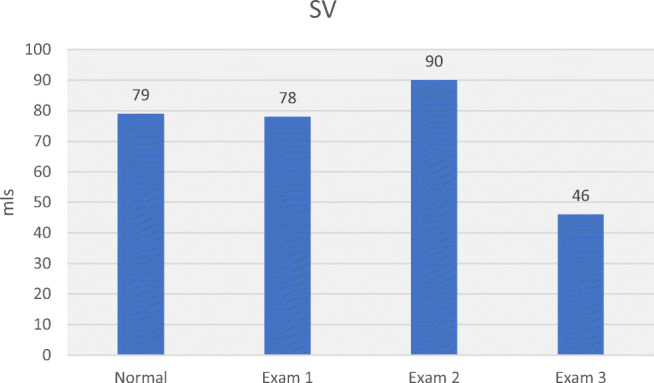
Normal SV, normal SV (Exam 1), mildly elevated SV in mild cardiogenic hypertension (Exam 2), and severe SV impairment, 2° to severely increased SVR in severe vasogenic hypertension (Exam 3) [19••]

**Fig. 7:**
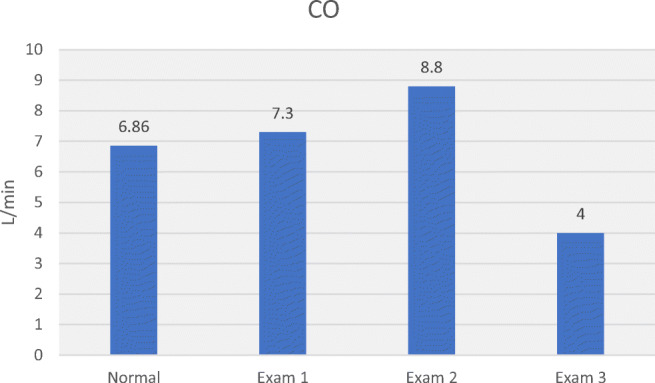
Normal CO, normal CO (Exam 1), mild to moderately elevated CO with mild cardiogenic hypertension (Exam 2) and severely impaired CO in severe vasogenic hypertension (Exam 3) [19••]

**Fig. 8:**
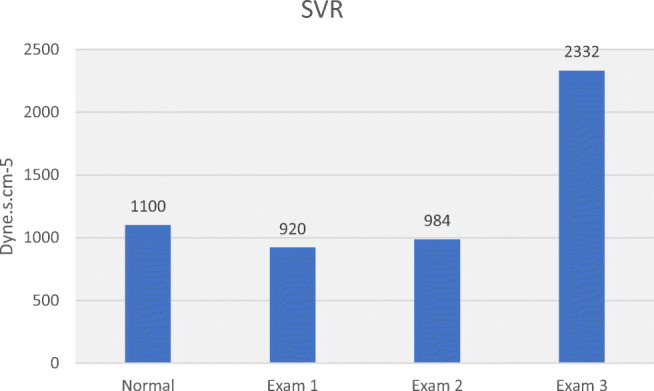
Normal reference SVR, low normal SVR in normal and mild cardiogenic hypertension (exams 1 and 2) and severely elevated in severe vasogenic hypertension (Exam 3) [19••]

**Fig. 9:**
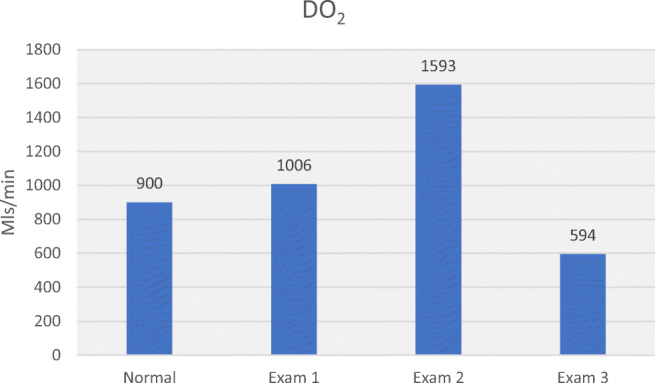
Normal reference DO_2_ in pregnancy, high normal DO_2_ in normal upregulation (exam 1), moderately increased DO_2_ in mild cardiogenic hypertension (Exam 2) and severely reduced DO_2_ in severe vasogenic hypertension (Exam 3) [19••]

**Fig. 10:**
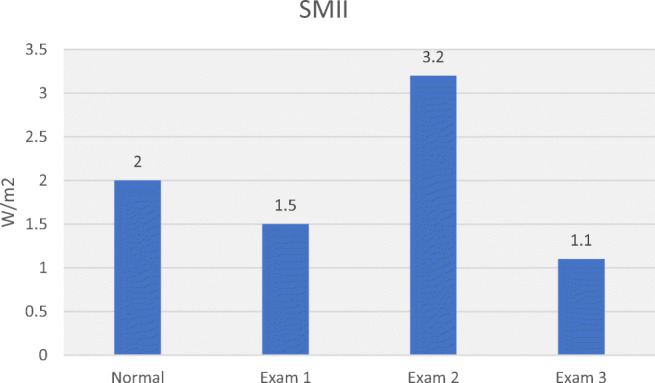
Normal reference SMII, low normal SMII in normal upregulated pregnancy (Exam 1), moderately increased SMII to deliver a 50% increased DO_2_ in mild cardiogenic hypertension (Exam2), and moderately reduced SMII in severe vasogenic hypertension (Exam 3) [19••]

Table 3 allows comparison of haemodynamic values in the 3 hypertensive subjects with normal values. Plotting MAP, SV, CO, SVR, SMII and DO_2_ for normal and hypertensive subjects (Figs. 5, 6, 7, 8, 9 and 10), demonstrates a unique haemodynamic pattern for each subject, and each parameter, and therefore a unique therapeutic approach. Neither SV, CO, SVR, SMII nor DO_2_, the therapeutic targets, correlated with MAP, the current method of defining and guiding therapy in maternal hypertension.

**Table 3:**
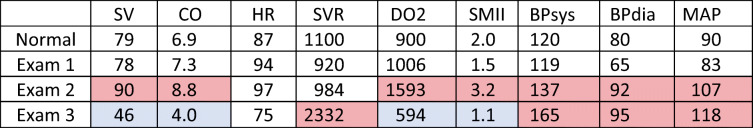
Normal BP and USCOM 1A haemodynamic reference values (19), and BP and haemodynamic findings in the three pregnant subjects with normal upregulated circulation (Exam 1), mild cardiogenic hypertension (Exam 2), and severe vasogenic hypertension complicated by severe ventricular failure (Exam 3). Red fill—parameter elevated, Blue fill—parameter decreased

#### Discussion

These three case studies of third trimester hypertensive pregnant subjects demonstrate the variability and unpredictability of maternal outcomes by BP, while abnormal haemodynamics more closely predicted outcomes (Table 1). Advanced haemodynamic monitoring of maternal circulation provided pathophysiologic understanding that was not provided by BP monitoring alone. The mathematical coupling of, and physiologic interdependence of, BP and cardiovascular function explains the systematic inadequacy of BP alone for guidance and management of maternal hypertension. First trimester haemodynamic monitoring may improve our understanding of the development of deranged maternal circulation, and the complex interaction of SV, CO, SVR, DO_2_ and SMII and their impact on maternal-foetal outcomes. This improved understanding may allow for early implementation of patient precise therapy.

#### Limitations

This observational study demonstrates the simple principles and application of advanced haemodynamic monitoring in identifying and managing maternal hypertension but is limited in its scope. Study of more subjects, randomised across a range of BPs, is required to better define the potential benefits of this physiologically guided approach. Further study of the temporal evolution of normal and pathological maternal haemodynamics may identify new personalised therapeutic approaches to its management and establish the utility of a routine first trimester scan.

## Conclusion

Advanced haemodynamic monitoring in pregnancy provides identification of circulatory pathophysiology at 5 to 11 weeks, and defines quantitative therapeutic targets not identified by BP alone. First trimester screening of SV, CO, SVR, SMII and DO_2_ may lead to an earlier and more accurate diagnosis, more precise therapy and improved outcomes in maternal hypertension.

## Abbreviations

BP: blood pressure in mmHg
MAP: mean arterial pressure in mmHg
SV: stroke volume in ml
CO: cardiac output in l/min
SVR: systemic vascular resistance in dyne s cm^−5^
HR: heart rate in beats/min
DO_2_: oxygen delivery in ml/min
VO2: oxygen consumption in ml/min
SMII: Smith Madigan Inotropy Index in W/m^2^
